# Bioconversion of coal to methane by microbial communities from soil and from an opencast mine in the Xilingol grassland of northeast China

**DOI:** 10.1186/s13068-019-1572-y

**Published:** 2019-10-08

**Authors:** Bobo Wang, Yanfen Wang, Xiaoyong Cui, Yiming Zhang, Zhisheng Yu

**Affiliations:** 1 College of Resources and Environment, University of Chinese Academy of Sciences, Beijing, 100049 People’s Republic of China; 2 College of Life Sciences, University of Chinese Academy of Sciences, Beijing, 100049 People’s Republic of China; 3 Beijing Municipal Ecological Environment Bureau, Beijing, 100048 People’s Republic of China

**Keywords:** Opencast coal mine, Coal, Grassland soil, Microbial community, Biomethane

## Abstract

**Background:**

The Xilingol grassland ecosystem has abundant superficial coal reserves. Opencast coal mining and burning of coal for electricity have caused a series of environmental challenges. Biogenic generation of methane from coal possesses the potential to improve economic and environmental outcomes of clean coal utilization. However, whether the microbes inhabiting the grassland soil have the functional potential to convert coal into biomethane is still unclear.

**Results:**

Microbial communities in an opencast coal mine and in grassland soil covering and surrounding this mine and their biomethane production potential were investigated by Hiseq sequencing and anaerobic cultivation. The microbial communities in covering soil showed high similarity to those in the surrounding soil, according to the pairwise weighted UniFrac distances matrix. The majority of bacterial communities in coal and soil samples belonged to the phyla *Firmicutes*, *Bacteroidetes*, *Actinobacteria* and *Proteobacteria*. The dominant bacterial genera in grassland soil included *Gaiella*, *Solirubrobacter*, *Sphingomonas* and *Streptomyces*; whereas, the most abundant genus in coal was *Pseudarthrobacter*. In soil, hydrogenotrophic *Methanobacterium* was the dominant methanogen, and this methanogen, along with acetoclastic *Methanosarcina* and methylotrophic *Methanomassiliicoccus,* was detected in coal. Network-like Venn diagram showed that an average of 28.7% of microbial communities in the samples belonged to shared genera, indicating that there is considerable microbial overlap between coal and soil samples. Potential degraders and methanogens in the soil efficiently stimulated methane formation from coal samples by the culturing-based approach. The maximum biogenic methane yields from coal degradation by the microbial community cultured from grassland soil reached 22.4 μmol after 28 day.

**Conclusion:**

The potential microbial coal degraders and methanogenic archaea in grassland soil were highly diverse. Significant amounts of biomethane were generated from coal by the addition of grassland soil microbial communities. The unique species present in grassland soil may contribute to efficient methanogenic coal bioconversion. This discovery not only contributes to a better understanding of global microbial biodiversity in coal mine environments, but also makes a contribution to our knowledge of the synthetic microbiology with regard to effective methanogenic microbial consortia for coal degradation.

## Background

Grasslands, comprising 37% of the terrestrial land area, are a major part of the global ecosystem [1]. Grasslands are typical multicomponent terrestrial ecosystems with different structures and distribution patterns across aboveground–underground gradient for microbial assemblages. Among contemporary ecosystems, the soil in a grassland system comprises diverse taxa estimated to range from thousands to millions [2, 3]. Owing to their peculiarities as ecosystems, grasslands not only represent a significant green ecological barrier, but also exhibit high microbial density and phylogenetic microbial diversity [2]. Microbial communities inhabiting grassland soils play an important role in adjusting and driving the biogeochemical cycle [4]. In addition to the abundant vegetation and soil resources, the grasslands of Inner Mongolia are extremely rich in superficial coal resources [5]. In terms of the quality and depth of the coal seams and the specific geological conditions at the site, coal mining processes are categorized into opencast and underground mining. According to the World Coal Institute (http://www.worldcoal.org), about 40% of global coal production involves opencast mining. The extracted coal is mostly used for the generation of electricity in many countries of the world. However, its by-products have caused alarming environmental and health hazards [6–8]. In recent years, the bioconversion of coal to methane or coal biogasification has been the research focus due to the environmental and industrial advantages involved [9, 10].

There have been several studies on potential coal degraders in underground coal mines containing biogenic methane, such as the Illinois Basin in the USA [11], the Gippsland Basin in Australia [12], the Ishikari coal field in northern Japan [13], the Alberta coal beds in western Canada [14] and the Eastern Ordos Basin in China [15]. Indigenous microbial communities inhabiting coalbed methane produced water can convert coal into methane in a synergistic manner [16]. In terms of the type of substrates available (acetate, C1 compounds and hydrogen/carbon dioxide), the most common methanogenic species can be categorized as acetoclastic, methylotrophic, and hydrogenotrophic [17, 18]. In addition, Mayumi et al. have reported that methoxylated aromatic compounds such as trimethoxy-cinnamate, trimethoxy-benzylalcohol, methoxy-benzoate, methoxy-phenol and trimethoxy-benzoate can also be used by *Methanomethylovorans* sp. to produce methane [19]. The dominant bacterial phyla include *Spirochetes*, *Firmicutes*, *Proteobacteria* and *Bacteroidetes* [20]. Furthermore, these dominant bacterial phyla and methanogens have been often detected in both biogenic and non-biogenic coalbed methane fields [21]. We et al. reported that the microbial communities in thermogenic gas coal mines exhibited potential for biogenic methanogenesis [22]. He et al. also reported that microbial communities obtained from non-production biogas coal mines could convert native lignite into biomethane [23]. Michiel et al. found that the microbial community in a non-producing Australian coal well had the functional potential to convert coal to methane [24]. However, compared with underground coal seam environments, little is known about the potential of coal bioconversion to methane from an opencast coal mine by indigenous microbial communities cultured from soils covering and surrounding this mine.

In China, more than 400 opencast coal mines are in operation with a coal production of 650,000,000 t/a. When coal seams are near the surface, it may be economical to extract the coal using opencast mining methods. Several studies have focused on the environmental problems caused by opencast coal mining, such as dust generation [25], changed topography [26] and heavy metal pollution [27]. These effects can be mitigated if the coal is utilized in situ into clean energy methane by methanogenic bioconversion. Thus, it is necessary to investigate the indigenous microbial communities inhabiting opencast coal seams and the surrounding environment, to modify the in situ conditions for potential generation of biomethane.

**Fig. 1:**
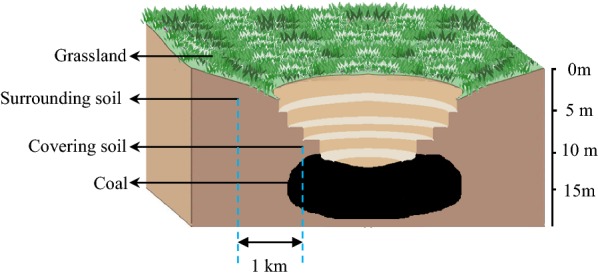
Profile sketch view of the opencast coal mine and the relative position of coal and soil samples

In this study, we investigated an opencast coal mine that was under mining operation in the Erlian basin, Xilingol grassland, China. The Xilingol grassland has abundant coal deposits [5]. Coal samples from the opencast coal mine and grassland soil samples covering and surrounding the coal mine were collected (Fig. 1). Molecular techniques combined with anaerobic cultivation were used to analyze the indigenous microbial communities inhabiting the coal and the grassland soil, and to determine their potential for biomethane generation. Microbial coal seam taxa between opencast coal mine examined herein and underground coal mine published previously were compared.

## Results

To investigate the potential of coal bioconversion to methane from an opencast coal mine by microbial communities cultured from grassland soil, the geochemical properties and microbial community characteristics of coal and grassland soil samples covering and surrounding the opencast coal mine were analyzed and performed as well as anaerobic cultivation under methanogenic conditions. The results indicated that the microbial communities cultured from the grassland soil covering or surrounding the opencast coal mine efficiently converted coal into biomethane.

### Properties of coal and soil

Coal from an opencast coal mine in the Erlian Basin contained a high proportion of carbon and a low proportion of nitrogen and sulfur (Table 1). Volatile matter and fixed carbon content were relatively high, up to 32.05% and 53.68%, respectively. The vitrinite and inertinite content in coal were quite high, whereas the mineral matter (0.31%) and liptinite (0.8%) content were relatively low. In addition, the coal sample had a reflectance (*R*_o,max_) of 0.31%. According to the standard of coal classification (GB5751-86) [28], coal samples from the opencast coal mine in this study were classified as lignite. A positive correlation was reported between liptinite content and methane production in the laboratory [29]; however, a negative correlation between coal rank and methane production has also been observed [20]. Thus, in light of the low liptinite content and low coal rank of the opencast coal mine in this study, it was necessary to investigate the methanogenic bioconversion potential of coal experimentally.

**Table 1:** Properties of coal sample in the opencast coal mine

Analysis	Yield (%)
Proximate analyses (air dried) %	
Moisture	7.5
Volatile matter	32.05
Fixed carbon	53.68
Ultimate analysis vol. (dry ash free) %	
Carbon	73.98
Hydrogen	4.09
Nitrogen	0.93
Sulfur	0.32
Oxygen	20.70
Petrographic analysis vol. %	
Vitrinite	24.6
Liptinite	3.5
Inertinite	71.6
Mineral matter	0.8
Reflectance (Ro, max) %	0.31
Classification	Lignite

The properties of grassland soil samples, including those from covering and surrounding soil are shown in Table 2. The collected three intra-group independent samples from either covering or surrounding soil in the Xilingol grassland showed similar properties with little variation. Covering soil NO_3_^-^-N, NH_4_^+^-N content and TOC were higher than those of surrounding soil. Soil pH and moisture content did not differ between covering and surrounding soil. TOC content was significantly higher than that in the soil from other opencast coal mines, such as the Antaibao coal mine [30]. High TOC is considered to be an indicator of bacterial activity [31, 32].

**Table 2:** Properties of grassland soil samples collected from covering and surrounding the opencast coal mine

Sample name^a^	NH_4_^+^–N (mg/g)	NO_3_^−^–N (mg/g)	Moisture content (%)	TOC (g/kg)	pH
CS1	0.029586	0.021268	4.83	8.25	7.9
CS2	0.024797	0.020909	4.45	8.28	7.9
CS3	0.023653	0.025826	4.65	9.33	8.1
SS1	0.022169	0.019433	4.57	7.67	7.8
SS2	0.021367	0.018375	4.73	7.82	7.9
SS3	0.021092	0.020548	4.69	7.37	7.8

*CS* covering soil, *SS* surrounding soil

### Detection of methanogenic potential of coal bioconversion

**Fig. 2:**
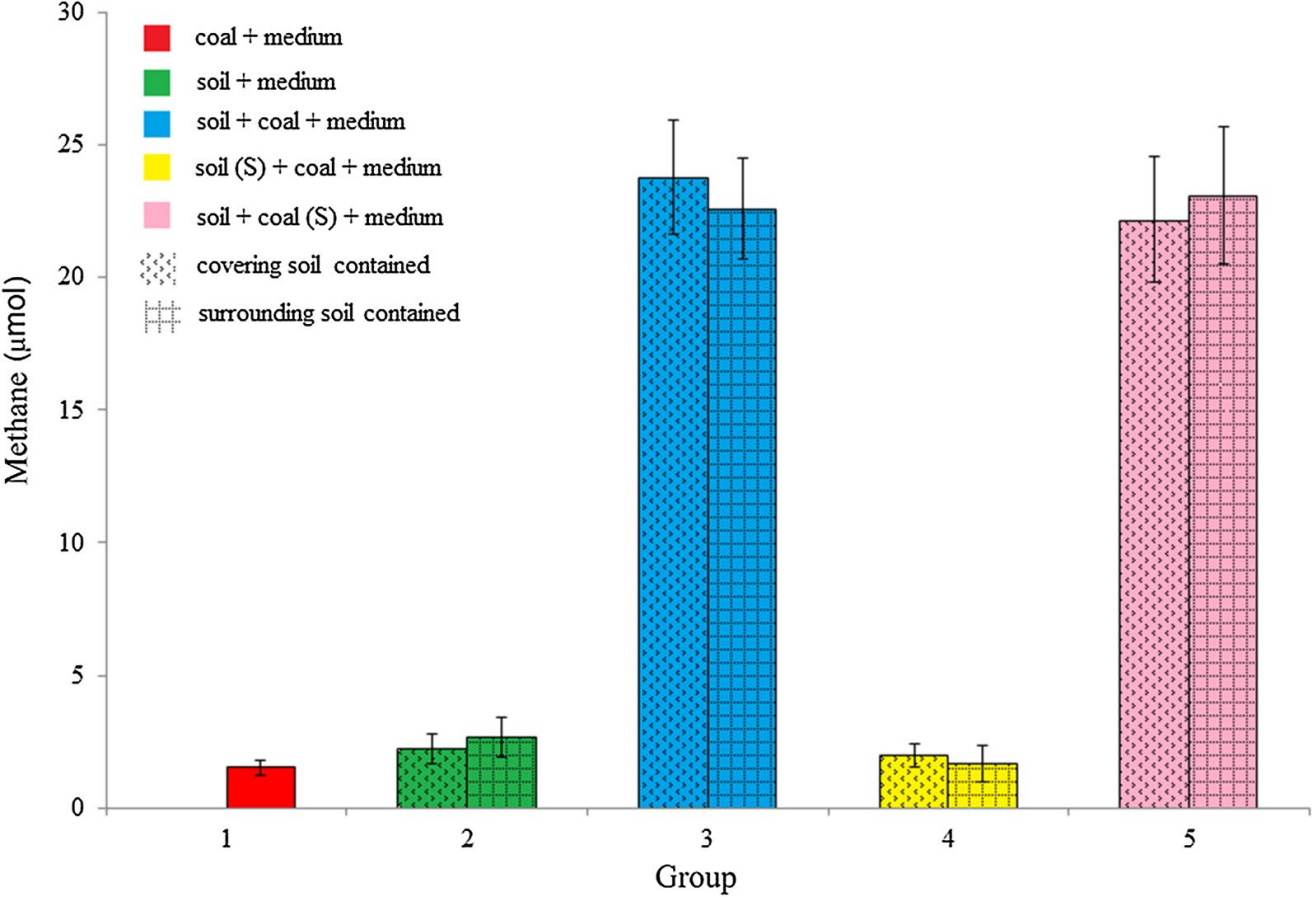
Plots of average methane production from coal bioconversion after the 28-day cultivation using coal and grassland soil microbial inoculums. Basal anaerobic medium was used for methanogenic coal bioconversion. Soil samples (0.5 g) and/or coal powder (0.3 g) were placed in 30 mL autoclaved anaerobic medium and incubated at 30 °C. Error bars represent standard deviation for replicates tubes. Soil (S) represents sterile soil; coal (S) represents sterile coal. Coal and soil represent the original coal and soil samples, respectively

To explore the biomethane production potential of coal from the opencast coal mine, five groups of cultures containing coal powder (0.3 g) and/or soil samples (0.5 g) as substrates were set up in the laboratory, while the microbial communities inhabiting the coal and/or soil were used for inoculation. Across the entire time course, significantly greater total yields of methane (*p* < 0.0001, Student’ s *t* test) were generated from the group containing 0.3-g coal and 0.5-g soil (either covering soil or surrounding soil) than from the first group containing 0.3-g coal alone or the second group containing 0.5-g coal alone (Fig. 2). There was no significant difference in methane production between cultures containing covering soil and cultures containing surrounding soil. Notably, a small amount of methane production was observed in group 2. The TOC content of the soil suggested the presence of carbon sources in the soil that could be utilized and converted into methane. However, the methane yields from soil (group 2) were considerably lower than those from soil and coal (group 3), suggesting that coal was the main carbon source for methane generation. Methane yields were low in group 4, which comprised 0.3-g coal and 0.5-g sterile soil (Fig. 2), while there was no significant difference between the methane yields in group 5 (containing 0.3-g sterile coal and 0.5-g soil) and those in group 3 (p > 0.05, Student’s *t* test). These results suggested that the microbial communities inhabiting the soil were the primary degraders of coal substrates.

### Sequence analysis by high-throughput sequencing

To elucidate the characteristics of the microbial communities inhabiting the grassland soil and opencast coal mine, the microbial communities in the operating opencast coal mine environment and surrounding grassland soil were thoroughly assessed via high-throughput sequencing. In total, 164,692 qualitative sequences were obtained from coal samples, 457,030 from covering soil samples, and 261,912 from surrounding soil samples. The average sequence lengths of bacteria and archaea were 253 and 383 base pairs, respectively. Q30 for bacteria and archaea ranged from 0.96 to 0.99, suggesting high sequencing accuracy. Additional details on the sequencing data are provided in Additional file 1.

### Microbial communities in coal from opencast coal mine

**Fig. 3:**
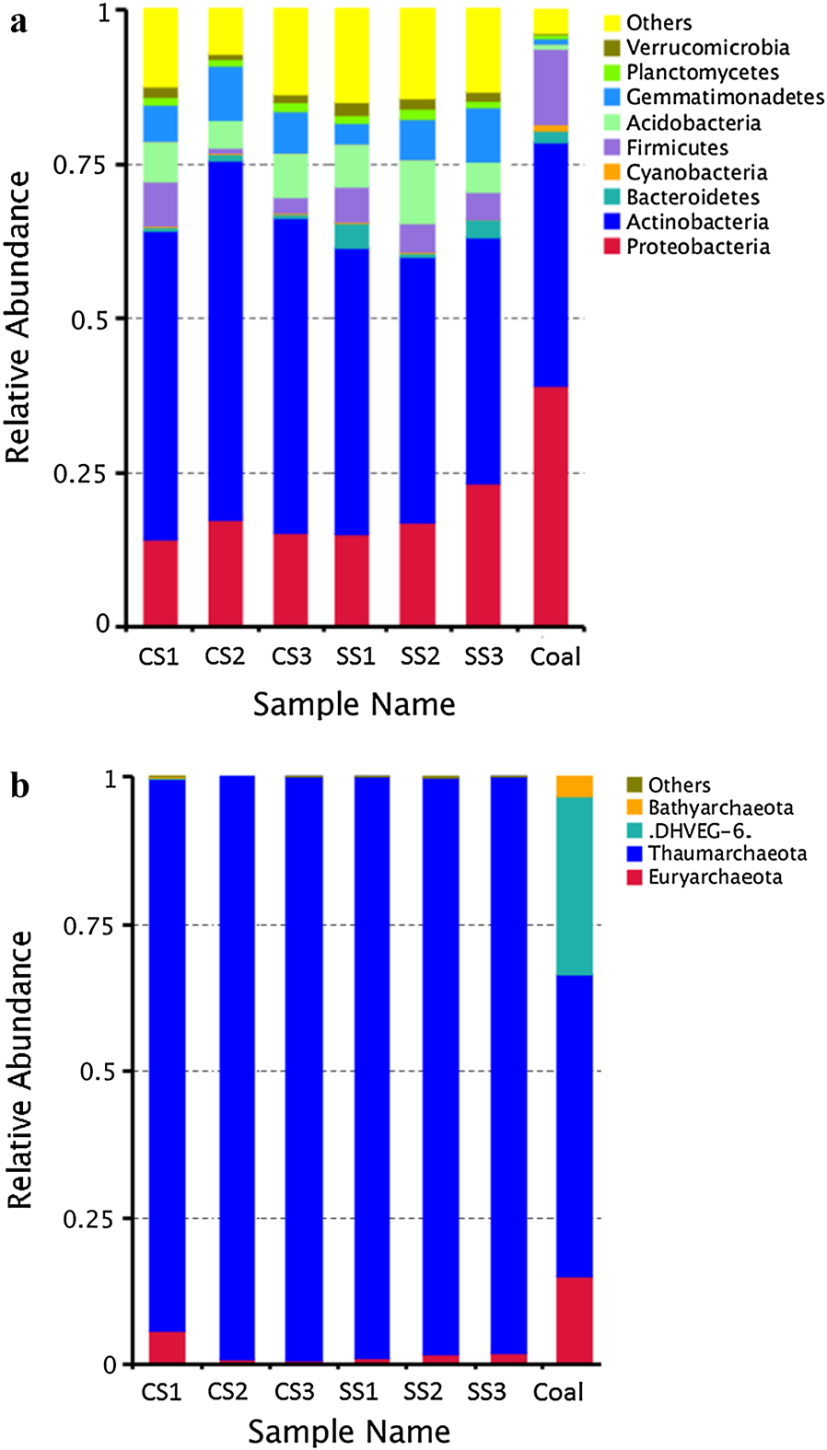
Relative abundance of 16S rRNA gene sequences in coal and soil samples at the phylum level from Illumina HiSeq sequencing for bacteria (**a**) and archaea (**b**). The phylum that constituted more than 1% of the bacterial and archaeal communities are shown in the figures. *CS* covering soil, *SS* surrounding soil

In coal samples, the relative abundance of *Actinobacteria* and *Proteobacteria* were 39.63% and 38.98% of all bacteria identified, respectively. Other primary bacteria identified in coal were members of *Firmicutes*, *Cyanobacteria* and *Bacteroidetes*, while members of *Acidobacteria*, *Gemmatimonadates*, *Planctomycetes*, and *Verrucomicrobia* were minor groups (Fig. 3a). The most abundant genus was *Pseudarthrobacter* (averaging 25.05% in coal), followed by *Brevundimonas*, *Methylotenera*, *Marinobacter* and *Parvibaculum*. Although *Thaumarchaeota* was the dominant archaeal phylum in coal samples as well as in soil samples, members of *Euryarchaeota*, *Woesearchaeota* and *Bathyarchaeota* were also major groups, accounting for a large proportion of the total archaeal community in coal. At the genus level, *Methanobacterium*, *Nitrososphaera* and *Methanosarcina* species accounted for a large portion of the total archaeal sequences in coal.

### Microbial communities in soil samples

The phylogenetic classification of microbial communities from soil samples is summarized in Fig. 3. Microbial comparison analyses based on pairwise weighted UniFrac distances matrix were performed to determine the similarities of microbial communities between covering soil and surrounding soil (Additional file 2). The average normalized Unifrac distances for bacterial and archaeal communities were 0.18 and 0.12, respectively, indicating there were high similarities of microbial communities between covering soil and surrounding soil (*p* > 0.05, Mantel test). Among the bacteria, *Actinobacteria* was the most abundant phylum in all six soil samples, accounting for an average of 53.06% and 43.1% of total bacteria in covering and surrounding soil samples, respectively. Other primary bacteria identified in the soil were members of *Proteobacteria*, *Firmicutes*, *Cyanobacteria*, *Acidobacteria*, *Gemmatimonadates*, *Planctomycetes* and *Verrucomicrobia*. The phylogenetic classification at genera level of archaea and bacteria for the soil and coal samples is listed in Additional file 3. The dominant genera included *Gaiella* (averaging 3.94% in covering soil and 2.60% in surrounding soil), *Solirubrobacter* (averaging 3.48% in covering soil and 2.16% in surrounding soil), *Sphingomonas*, and *Streptomyces*. In the archaeal communities, *Thaumarchaeota* was the most abundant phylum in all six soil samples, accounting for an average of 97.46% and 98.42% of the total archaea in covering soil and surrounding soil samples, respectively. Members of *Euryarchaeota* (averaging 2.40% in covering soil samples and 1.51% in surrounding soil samples) were also detected but constituted a minor fraction. The *Nitrososphaera* were the dominant archaea, comprising an average of 16.67% of the sequence reads at the genus level in the six soil samples. Genus *Methanobacterium* (averaging 0.27% in covering soil and 0.14% in surrounding soil) was a minor component in the soil samples.

### Comparison of the bacterial communities in coal and soil samples

**Fig. 4:**
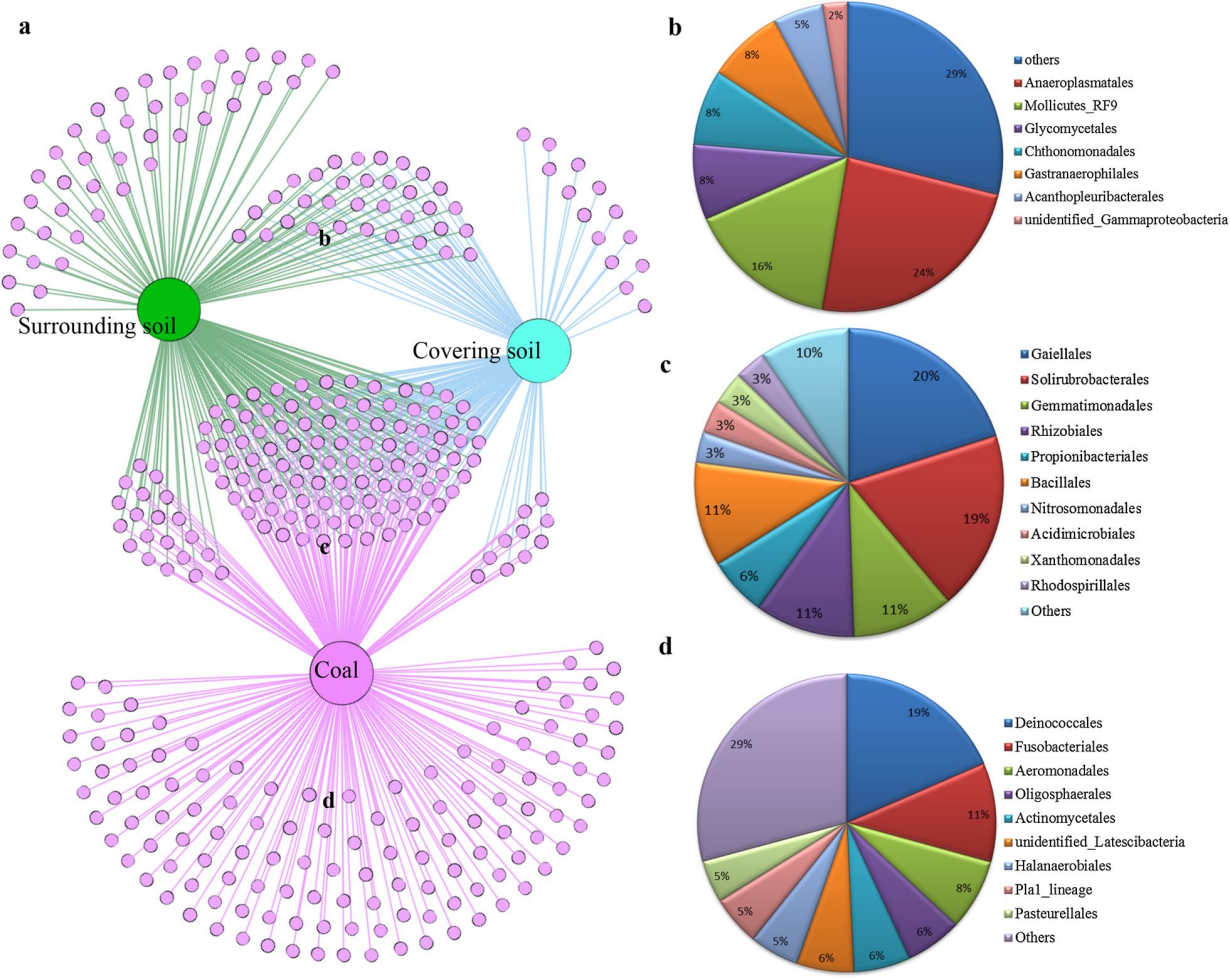
Network-like Venn diagrams of shared and unique genera in three habitats (**a**) and the distribution at order level of shared or unique genera in three habitats; the shared genera between covering soil and surrounding soil (**b**), the shared genera between soil and coal (**c**), and the unique genera in coal (**d**)

A network-like Venn diagram of bacterial communities showed overlap and partitioning of the microbial genera among coal, covering soil and surrounding soil (Fig. 4). Specifically, 28.7% of the bacterial genera were observed to overlap in these three different habitats. Bacterial genera *Gaiella* and *Solirubrobacter* contributed the majority of the sequences in the bacterial communities of the three habitats, followed by the genera *Streptomyces*, *Rubrobacter* and *Bacillus*. These genera were distributed in the orders *Gaiellales*, *Solirubrobacterales*, *Gemmatimonadales*, *Rhizobiales*, *Propionibacteriales*, *Bacillales* and so on (Fig. 4c). Unique genera in covering and surrounding soil comprised a minority of detected bacteria in the grassland soil, accounting for 4.1% and 14.6% of total bacterial sequences, respectively. In contrast, coal harbored the largest number of unique bacteria. *Corynebacterium*, *Propionibacterium*, *Lawsonella*, *Acidovorax* and *Azoarcus* were identified to be dominant unique bacteria genera in coal. Further analysis showed that 124 genera were clustered in the orders *Deinococcales*, *Fusobacteriales*, *Aeromonadales*, *Oligosphaerales*, *Actinomycetales*, *unidentified_Latescibacteria*, *Halanaerobiales* and *Pasteurellales*, comprising the majority of the unique prokaryotic sequences in coal (Fig. 4d). Unique microorganisms were observed to comprise only a small proportion in covering soil, probably owing to the location of the covering soil in the middle of the three habitats in the vertical direction.

### Phylogenetic analysis of representative OTUs with reference to coal bioconversion

**Fig. 5:**
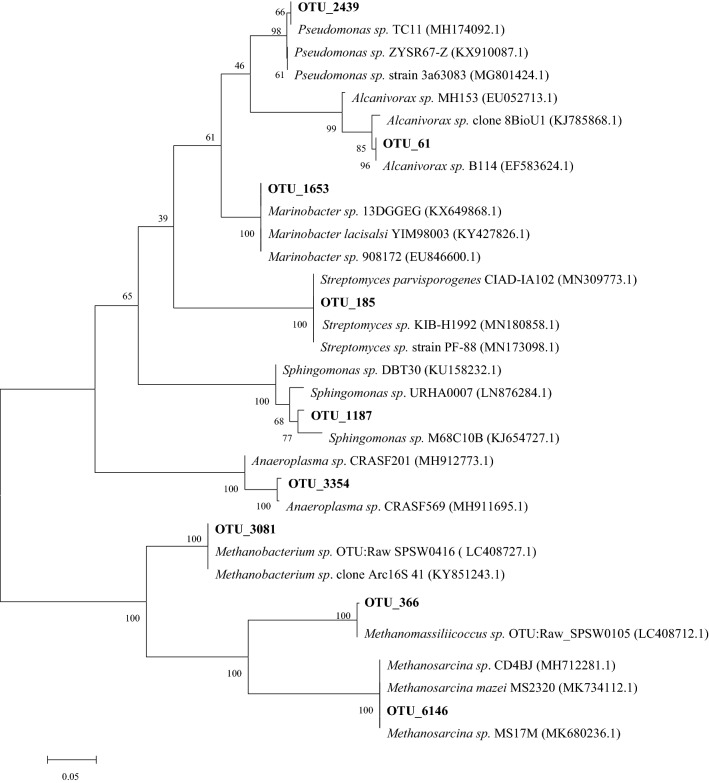
Phylogenetic tree for representative bacterial and archaeal OTUs (Boldface) with reference to coal bioconversion. The tree is based on maximum likelihood. The closest representative to each sequence is shown in parentheses (Accession Number). Numbers represent bootstrap values (100 trials). Bar, 5% estimated sequence divergence

To explore the potential synergistic microorganisms involved in the methanogenic process of coal from opencast coal mine, those OTUs similar to the widely reported microorganisms which are involved in the direct or indirect degradation of coal were selected as representative sequencing reads to construct phylogenetic tree. The phylogenetic tree illustrated the relationships between the representative bacterial and archaeal OTUs and their most similar sequences from the NCBI database with reference to coal biodegradation (Fig. 5). OTU_185 was closely related to the 16S rRNA gene from the *Streptomyces* sp. which can degrade lignite and bituminous coals [33]. OTU_3354 showed high match to the 16S rRNA gene from *Anaeroplasma* sp. which can utilize sterol contents in coal [34]. OTU_1653 showed close match to the 16S rRNA gene from *Marinobacter* sp. which is an efficient degrader of aliphatic and polycyclic aromatics [35]. OTU_2439 presented the close match to the 16S rRNA gene from *Pseudomonas* sp. which could degrade naphthalene, polyaromatics and alkane [36]. OTU_61 was closely related to the 16S rRNA gene from *Alcanivorax* sp. which is able to degrade broader alkanes [37]. OTU_3081 was closely related to the 16S rRNA gene from *Methanobacterium* sp. which is hydrogenotrophic methanogen. OTU_6146 showed close match to *Methanosarcina* sp. which have a high level of acetoclastic methanogenesis [38]. In addition to the above, some taxa identified in this study were also likely to be associated with coal biodegradation such as OTU_2, OTU_679, OTU_80, OTU_744 and OTU_949. OTU_2 was closely related to *Brevundimonas* sp. which is involved in the synergistic biodegradation of various hydrocarbons such as crude oil and sulfonamide compounds [39]. OTU_679 showed high match to the 16S rRNA gene from *Ferruginibacter* sp. which could remove aromatic pollutants such as quinoline and phenol in wastewater treatment process [40]. OTU_80, OTU_744 and OTU_949 belonging to the order *Clostridiales* has been suggested to play an important role in the initial stage of methanogenic coal bioconversion [41]. However, their definite biological functions in coal degradation are not clear yet. In addition, some aerobic and autotrophic taxa in this study were unlikely involved in the coal degradation, such as OTU_13, OTU_49, OTU_652, OTU_304 and OTU_3951. OTU_13 and OTU_652 were closely related to the *Methylomonas* sp. which is involved in methane oxidation process [42]. OTU_49, OTU_304 and OTU_3951 showed close match to *Candidatus_Nitrososphaera* sp. which is an obligate autotrophic microbe mainly involved in ammonia-oxidizing [43].

## Discussion

The majority of the previous studies on indigenous microbial community structures of coal and formation water were focused on biogenic or thermogenic methane-containing coal mines [21, 23, 44, 45]. This study provides direct evidence that there are diverse potential microbial coal degraders and methanogens inhabiting opencast coal mine environment, and microbial communities cultured from the grassland soil covering the coal or surrounding the coal mine can efficiently convert the coal into methane with a maximum yield rate reached 74.67 μmol/g coal, which is quite consistent with the methanogenic rate from lignite in the laboratory [20].

The final methane production from coal in the anaerobic cultures may be related to the properties of coal and soil. The methane production from coal from the opencast coal mine was at an intermediate yield level compared with observations recorded previously (ranging from 20.1 μmol/g coal to 162 μmol/g coal) [46, 47]. Scott et al. identified a general positive relationship between liptinite content and methane content [29]. Strąpoć et al. suggested that liptinite in coal is more prone to microbial degradation than vitrinite or inertinite [20]. Thus, the low liptinite content in coal from the opencast coal mine may be the main factor limiting methane production in this study. It is worth noting that there were no significant differences between methane productions of the groups containing covering soil and the groups containing surrounding soil (*p* < 0.05, Student’s *t* test). Extensive studies have proved that pH and moisture are important factors significantly influencing soil microbial community composition [48, 49]. In this study, there were no significant differences between pH or moisture contents of the soil samples collected from covering soil and surrounding soil. This might explain why the covering and surrounding soils shared similar microbial communities and there were no obvious differences between methane productions of the groups containing either covering or surrounding soils.

Illumina HiSeq sequencing revealed diverse potential microbial coal degraders inhabiting the grassland soil. Some dominant phyla, such as *Firmicutes*, *Bacteroidetes*, *Actinobacteria*, and *Proteobacteria* were observed to be involved in coal degradation and fueling methanogens to generate biomethane. These bacterial communities were often detected in the coalbed methane production water, situated deep underground [20]. The presence of these bacterial communities was an interesting and significant result, indicating the biodegradation potential of coal in an opencast coal mine. In addition, *Acidobacteria* (averaging 6.61% in soil) also accounted for a large fraction in terms of the total bacterial sequences. *Acidobacteria* has been shown to be a major phylum in coal mine wastewater treatment plants [50]. The genome analysis of *Acidobacteria* revealed that they make a significant contribution to the degradation of complex polymers [51]. Wegner et al. found that *Acidobacteria* have the competence to breakdown cellulose and xylan and metabolize lignin derivatives [52]. *Sphingomonas*, belonging to *Proteobacteria,* are capable of degrading polycyclic aromatic hydrocarbons [53]. *Streptomyces* belonging to *Actinobacteria* can degrade lignite and bituminous coals by secreting alkaline products [33]. The coal matrix consists of a complex mixture of lignin-derived macromolecules, aliphatic and aromatic hydrocarbons [20]. Thus, the dominant bacteria aforementioned might be able to degrade coal under suitable conditions.

The diversity of archaeal communities in coal was higher than that in soil. Hydrogenotrophic *Methanobacterium* (averaging 0.21% of total archaea in soil) was the dominant methanogen in soil. Interestingly, acetoclastic *Methanosarcina* (4.94% of total archaea in coal) and methylotrophic *Methanomassiliicoccus* (0.41% of total archaea in coal) were also detected in coal. The difference in archaeal communities between coal and soil suggested a significant degree of habitat preference for these microorganisms. Notably, while *Methanobacterium* and *Methanosarcina* were widely detected in the coal mine, *Methanomassiliicoccus* were rarely reported in coal seam environments except an abandoned coal mine in the United Kingdom [54]. The presence of these methanogens suggested a potential for biogenic methanogenesis under suitable temperature and anaerobic conditions. In addition, high levels of *DHVEG*-*6* (Deep-sea Hydrothermal Vent Euryarchaeotic Group 6, 30.1% of archaea) were detected in coal. *DHVEG*-*6* has been detected in municipal waste water treating methanogenic bioreactors, coal-bearing sediments, and deep-sea methane seep sediments [55, 56]. However, the metabolic and physiological functions of *DHVEG*-*6* are not clear. Nunoura et al. suggested that the *DHVEG*-*6* is likely to be heterotrophic archaea [57]. Although archaea, especially the methanogens, were more diverse in coal than in soil, methane yields in the groups containing coal microbes were much lower than those in the groups containing soil microbes. This is probably due to the difference between coal and soil bacterial community structures.

Microbial communities inhabiting the soil in the Xilingol grassland can effectively convert coal into biomethane. The potential degraders (e.g., *Firmicutes*, *Bacteroidetes*, *Actinobacteria* and *Proteobacteria*) and methanogens (*Methanobacterium*) in the soil may contribute to the formation of methane in a synergistic manner. Interestingly, some potential microbial degraders and methanogens were also present in coal; however, the methane production due to coal degradation by coal-inhabiting microorganisms was far less than that produced by the grassland soil cultures. The unique microorganisms inhabiting grassland soil may play an important role in the further degradation of coal. A Venn diagram intuitively revealed the unique bacteria shared between covering soil and the surrounding soil. At the order level, the distribution of these unique sequences in soil was dominated by *Anaeroplasmatales*, *Mollicutes_RF9*, *Glycomycetales*, *Chthonomonadales*, *Gastranaerophilales* (Fig. 4b). The distribution of unique OTUs in coal was dominated by *Deinococcales*, *Fusobacteriales*, *Aeromonadales* and *Oligosphaerales*. The order *Deinococcales* is often found in conditions of cold temperatures, ultraviolet radiation, desiccation and other extreme conditions [58]. Members of the order *Fusobacteriales* are suspected to be human pathogens causing intestinal inflammation [59]. The genus *Anaeroplasma* found in soil, belonging to *Anaeroplasmatales*, is a strictly anaerobic sterol-utilizing bacteria [34]. The sterol contents in different types of lignite deposits have been studied widely [60]. The sterol contents in coal might be further utilized by *Anaeroplasma* in soil. The genus *Chthonomonas* found in soil, belonging to *Chthonomonadales*, utilizes a variety of carbohydrate substrates as carbon and energy sources; *Chthonomonas* could grow synergistically with other cellulolytic microorganisms for carbohydrate substrates utilization and could hydrolyze hemicellulosic polymers immediately [61]. As the vitrinite of coal is derived from cellulose and lignin of plant tissues [20], it can be hypothesized that *Chthonomonas* may play an intermediate role in the degradation of coal. *Gastranaerophilales* are fermentative bacteria with the potential to produce formate, lactate, ethanol and CO_2_ under anoxic conditions [62]. *Gastranaerophilales* may also produce hydrogen synergistically while living with methanogens [62], as hydrogenotrophic *Methanobacterium* were simultaneously detected in the soil. The unique microbes in soil seem to exhibit a higher potential for coal degradation than those in coal. The metabolic characteristics and roles of these unique microbes involved in coal biodegradation still need to be verified by metagenomics and metatranscriptomics.

**Fig. 6:**
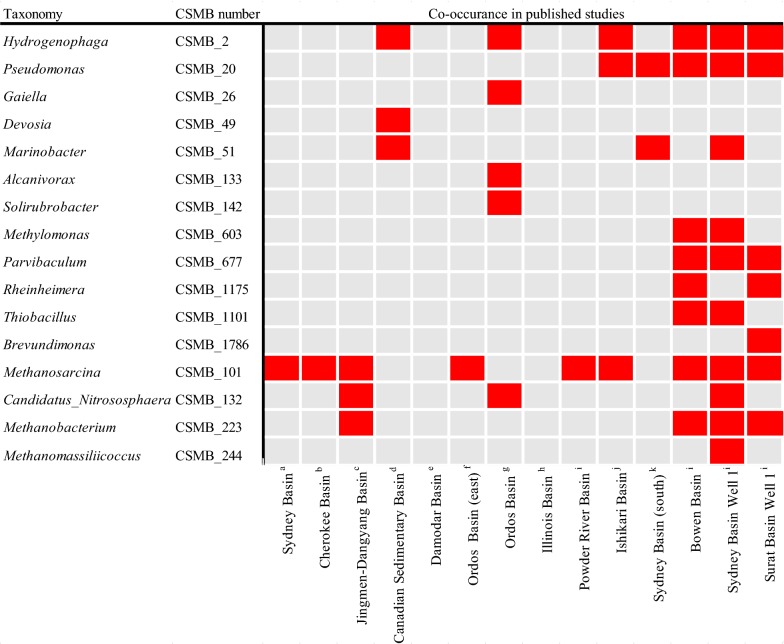
The abundant taxa from the opencast coal mine environment with their assigned CSMB numbers. The red square indicates the corresponding taxa found in this study had 98% or higher identity to the sequences in CSMB reference set and co-occurred across a range of published studies. **a** [80], **b** [45], **c** [22], **d** [14], **e** [81], **f** [15], **g** [82], **h** [11], **i** [83], **j** [13], **k** [84], **l** [63]

Coal Seam Microbiome (CSMB) is a dataset of OTU sequences specific to the coal seam environment, providing easy cross-sample comparisons for 16S rRNA gene amplicon data of microbial communities from various coal seam environments [63]. Some representative OTUs of the dominant genera in the opencast coal mine environment matched to the CSMB reference set also co-occurred in many underground coal seams across some regions of the world, as shown in Fig. 6. Nevertheless, none of the dominant OTUs matched in the CSMB reference set co-occurred in the Damodar basin or in the Illinois Basin. Of the co-occurring microorganisms, the identified methanogen *Methanosarcina* (CSMB_101) from the opencast coal mine has been observed in most of the coal seam environments. In addition, *Pseudomonas* (CSMB_20), a commonly observed genus in coal seam environments has also been found in the Ishikari, Bowen, Sydney and the Surat basin. *Pseudomonas* could degrade naphthalene, polyaromatics, and alkane [36, 64]. Members of the *Hydrogenophaga* (CSMB_2) observed in the Sydney, Surat, Ordos and the Bowen basin have shown the capacity to degrade xenobiotic compounds [65]. The *Proteobacteria* phylum members, *Marinobacter* (CSMB_51), observed in the Sydney basin and the Canadian sedimentary basin, has been reported to be efficient degraders of aliphatic and polycyclic aromatics [35]. The genera *Gaiella* (CSMB_26), *Alcanivorax* (CSMB_133) and *Solirubrobacter* (CSMB_142) only co-occurred in the Ordos basin. The *Proteobacteria* phylum members, *Parvibaculum* (CSMB_677), observed in the Sydney, Bowen and the Surat basins, possess an alkane oxidizing cytochrome P450-like protein to degrade alkanes, the most abundant components in coal [66, 67]. Interestingly, several dominant genera could not be mapped to the CSMB set. That is, *Sediminibacterium* (CSMB_2776) belonging to the Bacteroidetes and *Mycobacterium* (CSMB_3411) belonging to the *Actinobacteria* were not mapped to any member of the CSMB set. These findings indicated that a large subset of microbes present in opencast coal mines may have limited distributions, whereas the species mapped to the CSMB set seems to have cosmopolitan distributions.

**Fig. 7:**
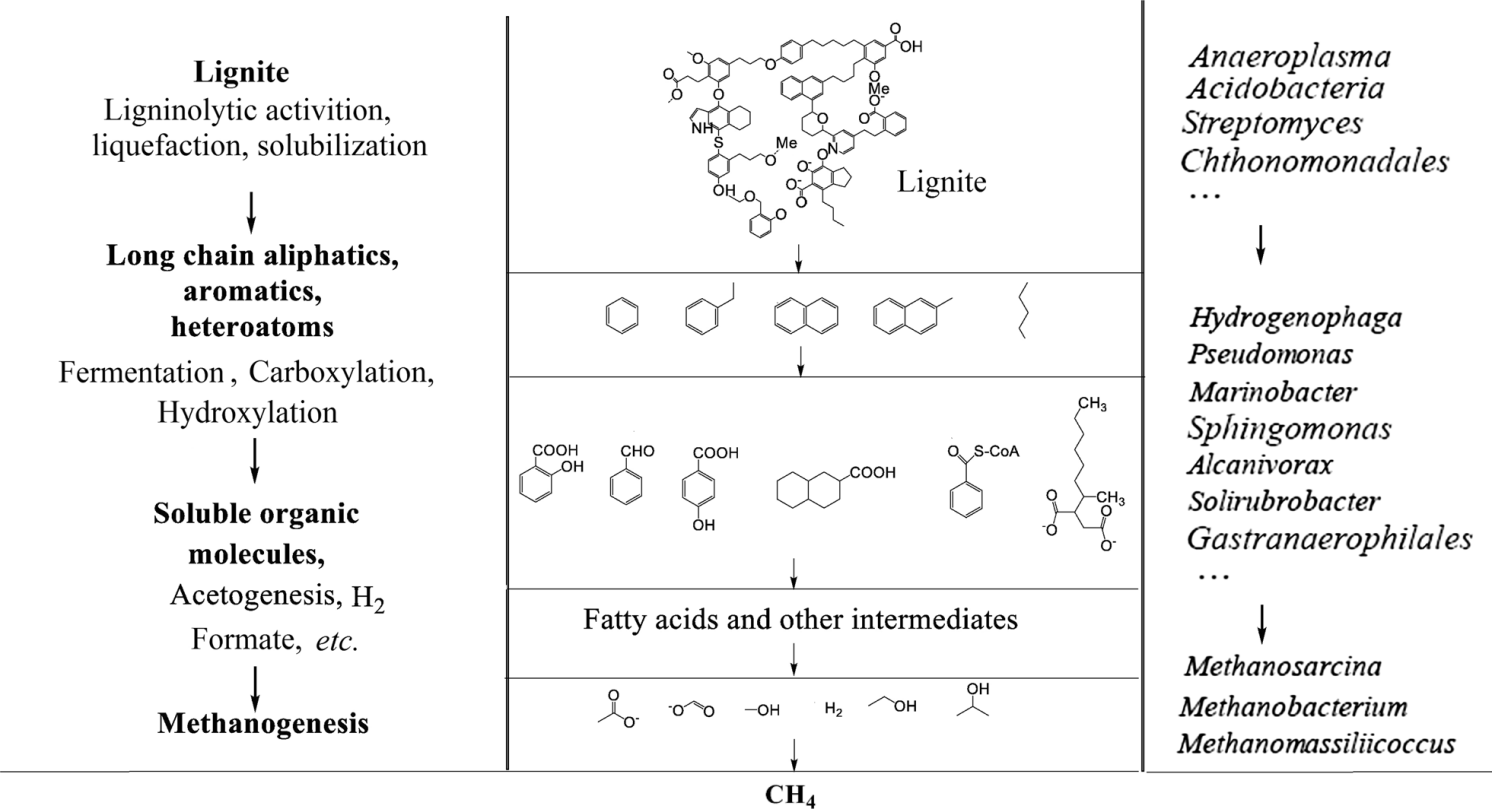
Proposed model of methanogenic coal biodegradation by the microorganisms observed in the opencast coal mine environment. A series of metabolic strategies are processed by a community of microorganisms under anoxic conditions (typical structure of lignite is adapted from Fakoussa and Hofrichter [85])

A model of methanogenic coal biodegradation by the microorganisms observed in this study is hypothesized based on the phylogenetic analysis of microbial communities and anaerobic cultivation (Fig. 7). Due to the recalcitrance, heterogeneity and hydrophobicity of the complicated coal matrix, the degradation of coal requires a series of metabolic strategies by a community of microorganisms under anoxic conditions [20]. At the initial degradation, the genera *Chthonomonas* and *Streptomyces* possess the capacity for the liquefaction and solubilization of coal. In addition, the genus *Anaeroplasma* could utilize sterol in coal immediately. After the initial degradation, complex macromolecular compounds, such as aromatics, heteroatoms and aliphatics, are activated by primary fermenting bacteria, including *Acidobacteria*. Then secondary fermenters, including *Pseudomonas*, *Alcanivorax*, *Gastranaerophilales*, *Sphingomonas* and *Hydrogenophaga*, have the capacity for complex compounds degradation to a variety of fatty acids, formate, lactate, acetate, ethanol and CO_2_. In addition, the release of biosurfactants by the genus *Pseudomonas* can promote the degradation of polycyclic aromatic hydrocarbons [68]. The final biodegradation products of ethylbenzene are potential substrates for hydrogenotrophic methanogens [68]. Benzoate is a central intermediate of many xenobiotic aromatic compounds in anaerobic degradation [69]. The biodegradation of per mol benzoate can produce 1.5 mol of acetate. The methyl group of acetate can be used by the genera *Methanosarcina* for methane formation [70]. However, due to the complicated interactions among the coal and the microbial communities, the definite metabolic flow from coal to methane needs further verification by the metabolomics analysis of optimized synthetic microbial communities.

## Conclusions

This is the first report on grassland soil microbial analysis regarding the degradation potential of coal from an opencast coal mine. The syntrophic bacterial and methanogenic archaeal communities in the grassland soil were highly diverse. Detectable biomethane was generated from coal by both the endogenous microbes in coal and by the addition of cultured grassland soil microbial communities. However, our microbial analysis coupled to anaerobic cultivation with coal revealed that microbial communities cultured from grassland soil effectively converted the coal into biomethane with maximum biogenic methane yields of 74.67 μmol/g coal. The similarity of pH and moisture contents in the covering and the surrounding soil may contribute to the similarity of microbial communities and methane production. The unique species belonging to the order *Anaeroplasmatales*, *Glycomycetales*, *Chthonomonadales* and *Gastranaerophilales*, etc. in soil may contribute to effective methanogenic coal bioconversion. These findings suggest that coal, even from the opencast coal mine, could be used to generate biogenic methane by soil microorganisms cultured from the adjacent grassland soil. Nonetheless, further studies are required to explore the interaction between bioenergy and environmental microorganisms to enhance biomethane production.

## Methods

### Description of sampling site, sample collection and physicochemical properties

The Erlian Basin located in Xilingol grassland is an intracontinental Cretaceous basin in north-east China, Inner Mongolia. It is enclosed by the Hailar Basin to the north, the Ordos Basin to the south-west, the Songliao Basin to the east, and the east Gobi Basin to the west [71]. Samples including coal and covering soil were collected from an in-operation opencast coal mine located in central Erlian Basin (Fig. 1). The average sampling depth was 10–15 m. For coal sampling, a big coal block about 2–3 kg was collected from the inside of newly exposed coal mining faces using a sterilized shovel and immediately placed into sterile valve bags and stored at 4 °C in an anaerobic chamber before culture. Three sampling locations were set to avoid sampling bias. Three grassland soil samples (named CS1, CS2 and CS3) covering adjacent coal seams and three surrounding grassland soil samples at a distance of 1 km from the coal mine (named SS1, SS2 and SS3) were collected and immediately sealed in a sterile valve bag and transported to the lab on dry ice.

The collected coal block was ground into powder using a sterile pestle and mortar and sieved through a 200-mesh sieve to collect particles less than 250 μm in diameter in an anaerobic operating chamber (XinmiaoYQX-11, China). The soil samples were thoroughly homogenized and sieved to ≤ 2-mm particle size for subsequent experiments. Samples used for culture were stored at 4 °C; samples used for microbial community analysis were frozen at − 80 °C until DNA extraction.

The physicochemical properties of the coal samples were determined at the Centre of the China Coal Research Institute. Soil moisture contents were measured by drying at 105 °C for 24 h. Soil NO_3_^−^-N and NH_4_^+^-N contents were analyzed using KCl solution extraction (soil mass/solution ratio was 5:1) and an auto-analyser (SEAL Analytical GmbH, Germany). A TOC Analyzer system (Liqui TOC II; Germany) was used to measure total organic carbon (TOC) contents in soils with the same extraction method. Soil pH was measured using a pH meter (FiveEasy Plus™, Mettler Toledo, Switzerland) with the soil mass–water ratio of 1:5.

### Detection of biomethane potential of the coal

Basal anaerobic medium, prepared as described previously [11], was used for methanogenic coal bioconversion. Soil samples (0.5 g) and/or coal powder (0.3 g) were placed into 30-mL autoclaved anaerobic medium in the 140-mL serum bottles according to the experimental design. All serum bottles were capped with butyl rubber stoppers and sealed by aluminum crimps. The headspace of the serum bottles was extracted in an anaerobic operating chamber and then degassed ten times with pure N_2_ to remove oxygen. Cultures were incubated in static condition at 35 °C and maintained in dark. To study the biomethane generation potential of coal, five groups of microcosms were established. The first group contained 0.3 g of ground coal and medium; the second group contained 0.5 g of soil and medium; the third group consisted of coal (0.3 g) + soil (0.5 g) + medium; the fourth group consisted of coal (0.3 g) + soil (0.5 g, sterile) + medium; the fifth group consisted of coal (0.3 g, sterile) + soil (0.5 g) + medium. Each group included three replicates. For the tests of sterile samples, powdered soil and coal were sterilized by freeze thawing five times using liquid nitrogen. Confirmation of sterilization was performed by serially diluting the powdered coal or soil samples, after freeze thawing, in sterile phosphate buffer, and plating the suspensions on LB agar plates, for incubation at 30 °C for 48 h and observation for microbial growth [72]. A gas chromatograph equipped with a flame ionization detector was used to detect the methane content in the headspace of the serum bottles weekly.

### DNA extraction and amplification

Genomic DNA from the soil samples was extracted using a FastDNA SPIN kit immediately (MP Biomedicals, USA) according to the manufacturer’s instructions. Coal samples were flushed using a phosphate buffer (0.05 M, pH 7.4), supplemented with 0.2% Tween 80 [15], and then extracted using a FastDNA SPIN kit according to the manufacturer’s instructions. The DNA was concentrated with Amicon Ultra centrifugal filter units (Millipore, MA, USA). Owing to the low DNA yield from coal samples, the microbial DNA samples used in this study were a mixture of three independent coal samples from the same coal mine. Particularly, this method could remove interindividual variability and highlight the integral microbial communities of coal from the opencast coal mine. For the Illumina HiSeq sequencing, bacterial libraries were created using the bacterial primer set: 515F: GTGCCAGCMGCCGCGG and primer 907R: CCGTCAATTCMTTTRAGTTT [73]. The archaeal libraries were obtained using the universal archaeal primer 519F: CAGCCGCCGCGGTAA and primer 915R: GTGCTCCCCCGCCAATTCCT [15]. In addition, to obtain accurate results, we conducted three independent PCRs for final DNA sample to minimize the impact of potential early round PCR errors [74], and then combined three independent PCR products to perform subsequent sequencing. Therefore, the results based on sequencing were presented as a final value considering the biological and technical replicates established before sequencing.

### Sequencing and phylogenetic analysis

The concentration of purified PCR products was monitored using a QuantiFluorTM-ST (Promega, USA). The DNA libraries were prepared with purified PCR products using a TruSeq^®^ DNA Sample Preparation Kit. Constructed DNA libraries were checked using qPCR and Qubit. Sequencing was performed on a HiSeq 2500 PE250 platform (Novogene Bio-tech, Co., Ltd. Beijing, China). Raw sequence data were trimmed and subjected to quality control evaluation with Qiime (V1.9.1, http://qiime.org/scripts/split_libraries_fastq.html) [75]. Sequences were clustered into operational taxonomic units (OTUs) at 97% sequence similarity using Uparse software (v7.0.1001) [76]. The representative sequences for OTUs were classified against the SSUrRNA sequence database [77]. In addition, the OTUs results of soil samples belonging to the same habitat were rarefied in terms for per habitat. The network-like Venn diagram was created using Cytoscape3.1.1 based on the merged OTUs [78]. Average pairwise weighted UniFrac distances between samples were used to determine the similarity of microbial communities. Mantel test were used to assess if differences in microbial community compositions were significant [79]. A phylogenetic tree was constructed in the MEGA 7.0 using the maximum likelihood.

### Nucleotide sequence accession number

The obtained sequences in this study were deposited in the NCBI short read archive (SRA) under Bioproject Accession Number PRJNA548311, with Biosample numbers SAMN12077157–SAMN12077163.

## Supplementary information

**Additional file 1: Table S1.** Detailed quality results of the sequencing data.

**Additional file 2: Table S2.** Weighted UniFrac distances matrix for microbial communities in covering and surrounding soil samples.

**Additional file 3: Table S3.** The phylogenetic classification at genera level of bacteria and archaea for the soil and coal samples.

## Data Availability

The datasets used and/or analyzed during the current study are available from the corresponding author on reasonable request.

## Abbreviations

CBM: coalbed methane
CSMB: Coal Seam Microbiome
CS: covering soil
SS: surrounding soil
OTU: operational taxonomic units
SRA: short read archive
